# Prevalence, Antimicrobial Resistance, and Molecular Characteristics of MRSA in Saudi Arabia: A Retrospective Study

**DOI:** 10.3390/microorganisms14010227

**Published:** 2026-01-19

**Authors:** Soha Abdallah Moursi, Mohd Saleem, Azharuddin Sajid Syed Khaja, Ehab Rakha, Kareemah Salem Alshurtan, Nahed Fathallah Fahmy, Amal Daher Alshammari, Emad Abboh Abdallah Abboh, Metab Nasser Alshammari, Homoud Almalaq

**Affiliations:** 1 Department of Pathology, College of Medicine, University of Hail, Hail 55476, Saudi Arabia; m.saleem@uoh.edu.sa (M.S.); skazharuddin@uoh.edu.sa (A.S.S.K.); e.abboh@uoh.edu.sa (E.A.A.A.); 2 Laboratory Department, King Khalid Hospital, Hail 55421, Saudi Arabia; ehabrakha@yahoo.com; 3 Clinical Pathology Department, Faculty of Medicine, Mansoura University, Mansoura 35516, Egypt; 4 Department of Internal Medicine, College of Medicine, University of Hail, Hail 55476, Saudi Arabia; k.alshurtan@uoh.edu.sa; 5 Department of Medical Microbiology and Immunology, Faculty of Medicine, Sohag University, Sohag 82524, Egypt; dr.nahedfahmy@gmail.com; 6 Department of Family Medicine, College of Medicine, University of Hail, Hail 55476, Saudi Arabia; amal.alshammari@uoh.edu.sa; 7 Department of Infection Control, King Khalid Hospital, Hail 55421, Saudi Arabia; metab2201@gmail.com; 8 Department of Medical Supplies, Hail Health Cluster, Hail 55471, Saudi Arabia; halmalaq@moh.gov.sa

**Keywords:** antimicrobial resistance (AMR), community-associated MRSA (CA-MRSA), healthcare-associated MRSA (HA-MRSA), methicillin-resistant *Staphylococcus aureus* (MRSA), molecular epidemiology

## Abstract

Methicillin-resistant *Staphylococcus aureus* (MRSA) is a significant pathogen in both healthcare-associated (HA-MRSA) and community-associated (CA-MRSA) infections, posing major challenges due to its evolving antimicrobial resistance (AMR) and genetic diversity. This study investigates the prevalence, antimicrobial resistance patterns, and molecular characteristics of HA-MRSA and CA-MRSA isolates in Saudi Arabia. A retrospective analysis was conducted on 178 MRSA isolates obtained from clinical samples. MRSA identification was performed using cefoxitin disk diffusion, and antimicrobial susceptibility testing for vancomycin, linezolid, and ciprofloxacin was conducted using the BD Phoenix M50 system. Molecular characterization included SCCmec typing, *spa* typing, and PCR-based detection of virulence genes (*pvl*, *tst*, *eta*, *etb*, *lukS*, *lukF*). Statistical analysis was carried out using SPSS, with a significance threshold of *p* < 0.05. Among 1496 *S. aureus* isolates, 178 (11.9%) were confirmed as MRSA, with HA-MRSA (61.8%) being more prevalent than CA-MRSA (38.2%). Notably, 7.8% of HA-MRSA isolates exhibited heteroresistant vancomycin-intermediate *S. aureus* (hVISA). Ciprofloxacin resistance was significantly higher in HA-MRSA (85.0%) compared to CA-MRSA (38.9%). SCCmec type V was the predominant genotype (87.1%), suggesting increased infiltration of CA-MRSA strains into hospital settings. *Spa* typing revealed high genetic diversity, with *t037* being the most common (27%). Virulence genes were detected in 6% of isolates, indicating limited dissemination of these factors. The findings highlight the increasing prevalence of MRSA, the emergence of hVISA, and shifts in clonal distribution, underscoring the need for ongoing molecular surveillance and stringent antimicrobial stewardship programs to control MRSA spread in both healthcare and community environments.

## 1. Introduction

Methicillin-resistant *Staphylococcus aureus* (MRSA) is a major global public health threat, causing severe infections in both hospital and community settings [1]. Traditionally, MRSA was categorized into healthcare-associated MRSA (HA-MRSA) and community-associated MRSA (CA-MRSA) based on epidemiological and molecular characteristics [2]. HA-MRSA strains are typically multidrug-resistant and linked to nosocomial infections, whereas CA-MRSA strains tend to be more virulent but less resistant to antibiotics [3]. However, recent evidence suggests an increasing overlap between HA-MRSA and CA-MRSA, with community strains being detected in hospitals and vice versa, making epidemiological differentiation more complex [4].

The rising antimicrobial resistance (AMR) in MRSA isolates presents a significant clinical challenge, particularly in Middle Eastern countries, where high antibiotic consumption has contributed to the emergence of resistant strains [5]. Vancomycin remains the gold standard for MRSA treatment, but heteroresistant vancomycin-intermediate *S. aureus* (hVISA) has been reported in several regions, raising concerns about treatment efficacy [6]. Additionally, fluoroquinolone and oxazolidinone resistance among MRSA isolates is increasing, necessitating continuous surveillance of antimicrobial susceptibility patterns [7].

Understanding the molecular characteristics of MRSA, including staphylococcal cassette chromosome mec (*SCCmec*) types, *spa* types, and virulence gene profiles, is essential for tracking MRSA evolution and developing effective infection control strategies [8]. *SCCmec* types I–III are commonly found in HA-MRSA, whereas *SCCmec* IV and V are predominant in CA-MRSA [9]. Similarly, *Spa* typing allows for clonal lineage identification, helping to determine the genetic diversity of circulating MRSA strains [10]. Virulence factors such as Panton-Valentine leukocidin (PVL), toxic shock syndrome toxin (*tst*), and exfoliative toxins (*eta*, *etb*) contribute to MRSA pathogenicity, making their detection critical in epidemiological studies [11].

Given the increasing burden of MRSA infections in Saudi Arabia, there is an urgent need to assess the prevalence, antimicrobial resistance patterns, and molecular characteristics of MRSA isolates from both hospital and community settings [12]. This study aims to (i) determine the prevalence of HA-MRSA and CA-MRSA, (ii) evaluate antibiotic susceptibility patterns, including vancomycin, linezolid, and ciprofloxacin resistance, and (iii) characterize *SCCmec* types, *spa* types, and virulence genes among MRSA isolates. Understanding these factors will provide critical insights for improving infection control policies and guiding antimicrobial stewardship programs in the region.

## 2. Materials and Methods

### 2.1. Study Design and Sample Collection

A retrospective study was conducted to determine the prevalence and molecular characteristics of Methicillin-Resistant *Staphylococcus aureus* (MRSA) in clinical isolates obtained from the Outpatient Department (OPD) and Inpatient Department (IPD) of King Khalid Hospital, Saudi Arabia. MRSA isolates from OPD cases were classified as CA-MRSA, while those from IPD cases were categorized as HA-MRSA. This study analyzed *S. aureus* isolates collected between 1 January 2023, and 26 November 2024, from King Khalid Hospital. Among a total of 1496 clinical samples that exhibited *S. aureus* growth, 178 isolates were confirmed as MRSA and included for further molecular characterization and analysis, whereas virulence determination was carried out in all isolates. To avoid duplication and overestimation of prevalence and resistance rates, only one isolate per patient was included in the analysis. Specifically, the first MRSA isolate recovered per patient during the study period was analyzed. Repeat isolates from the same patient obtained within the same clinical episode or within a 30-day period were excluded.

#### Ethical Approval

This research project adhered to the ethical guidelines of the University of Hail, KSA (IRB/KSA: H-08-L-074) and University of Hail (H-2023-390), Saudi Arabia. Participant confidentiality was maintained by using unique identifying codes to protect personal data.

### 2.2. Bacterial Isolation and Identification

In this retrospective study, clinical specimens were cultured on mannitol salt agar and 5% sheep blood agar and incubated at 37 °C for 24–48 h under aerobic conditions. *Staphylococcus aureus* isolates were initially identified based on colony morphology, Gram staining, catalase test, and slide coagulase test. Further confirmation was performed using the tube coagulase test and DNase test. Methicillin resistance was determined phenotypically using cefoxitin disk diffusion and the BD Phoenix M50 system (Franklin Lakes, NJ, USA) in accordance with CLSI guidelines. Routine PCR detection of the *mecA* and *mecC* genes was not performed for all isolates. However, molecular characterization by SCCmec typing was conducted on confirmed MRSA isolates, which indirectly indicates the presence of the mecA-mediated resistance determinant.

### 2.3. Antimicrobial Susceptibility Testing (AST)

Antibiotic susceptibility testing was conducted by Phoenix M50 system, following CLSI guidelines [13]. The following antibiotics were tested, (i) vancomycin (glycopeptide), (ii) linezolid (oxazolidinone), and (iii) ciprofloxacin (fluoroquinolone). For vancomycin, minimum inhibitory concentrations (MICs) were determined using the Etest method. Vancomycin susceptibility was interpreted solely based on MIC values obtained by E-test. Heteroresistant vancomycin-intermediate *Staphylococcus aureus* (hVISA) was screened using the Etest macro-method and confirmed with population analysis profiling-area under the curve (PAP-AUC) testing [14]. For linezolid and ciprofloxacin, MIC values were determined using broth microdilution, and the results were categorized into MIC50 (minimum concentration inhibiting 50% of isolates) and MIC90 (minimum concentration inhibiting 90% of isolates) [15]. The median MIC and interquartile range (IQR) were also calculated. Quality control was performed using *S. aureus* ATCC 29213 and *Enterococcus faecalis* ATCC 51299 as reference strains [16].

### 2.4. Molecular Characterization

#### 2.4.1. DNA Extraction

Genomic DNA was extracted from MRSA isolates using the QIAamp DNA Mini Kit (Qiagen, Hilden, Germany) following the manufacturer’s protocol. DNA purity and concentration were assessed using NanoDrop spectrophotometry (Thermo Fisher Scientific, Waltham, MA, USA).

#### 2.4.2. Detection of SCCmec Types

The staphylococcal cassette chromosome mec (*SCCmec*) types were identified using multiplex PCR with specific primers targeting *SCCmec* Types I, II, III, IV, and V [17]. PCR amplification was performed in a Bio-Rad T100 Thermal Cycler (Bio-Rad, Hercules, CA, USA) under following optimized cycling conditions: initial denaturation at 95 °C for 5 min, followed by 35 cycles of denaturation at 95 °C for 30 s, annealing at 55–58 °C (depending on the target gene) for 30 s, extension at 72 °C for 45 s, and a final extension at 72 °C for 7 min.

#### 2.4.3. Spa Typing

Molecular epidemiology was assessed through *spa* typing using specific primers targeting the polymorphic X region of the *spa* gene [18]. PCR products were sequenced using the Sanger sequencing method, and sequence data were analyzed using the Ridom StaphType software (Version 2) to determine *spa* types [19].

#### 2.4.4. Detection of Virulence and Resistance Genes

The presence of Panton-Valentine leukocidin (PVL), toxic shock syndrome toxin (*tst*), exfoliative toxins (*eta* and *etb*), and leukocidin genes (*lukS* and *lukF*) was determined using PCR-based methods [20]. PCR conditions included an initial denaturation at 95 °C for 5 min, followed by 35 cycles of denaturation (95 °C for 30 s), annealing (varied by gene target), and extension (72 °C for 1 min), with a final extension at 72 °C for 5 min. PCR products were visualized on 1.5% agarose gels stained with ethidium bromide [21].

### 2.5. Statistical Analysis

Data were analyzed using SPSS version 25 (IBM Corp., Armonk, NY, USA) [22]. Descriptive statistics, including means, medians, standard deviations (SD), interquartile ranges (IQR), MIC50, and MIC90 values, were computed for MIC distributions. For categorical variables (e.g., *SCCmec* types, *spa* types, virulence gene presence), Chi-square or Fisher’s exact tests were used. Differences in MIC values between HA-MRSA and CA-MRSA isolates were assessed using the Mann–Whitney U test. A *p*-value < 0.05 was considered statistically significant [23]. All analyses were performed on a per-patient basis, with each isolate representing a unique patient.

## 3. Results

### 3.1. Antimicrobial Resistance Trends and SCCmec

**Figure 1:**
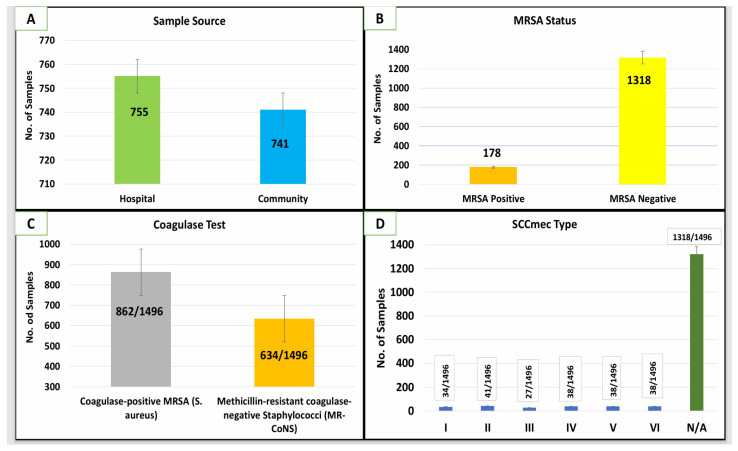
Distribution and Characterization of MRSA Isolates from Hospital and Community Sources in the Kingdom of Saudi Arabia. Bar charts represent the distribution and characteristics of MRSA isolates (n = 178) from both hospital and community settings in Saudi Arabia. (**A**) Shows the distribution of MRSA isolates based on the sample source, with a higher prevalence in hospital-derived samples compared to community sources. (**B**) Depicts the MRSA status among the total samples (n = 1496), highlighting a predominance of MRSA-negative samples. (**C**) Illustrates Coagulase test among methicillin-resistant staphylococcal isolates, distinguishing confirmed MRSA (coagulase-positive *S. aureus*) from methicillin-resistant coagulase-negative staphylococci (MR-CoNS). (**D**) Displays the distribution of *SCCmec* types among MRSA isolates, indicating the dominance of *SCCmec* Type V.

The distribution and characterization of MRSA isolates are illustrated through four bar charts in Figure 1A–D. Figure 1A depicts the distribution of MRSA isolates based on the sample source, revealing that hospital-derived MRSA isolates (n = 110, 61.8%) significantly outnumber those from community sources (n = 68, 38.2%). This suggests a higher burden of MRSA within hospital settings, likely reflecting the increased risk factors associated with healthcare environments, such as prolonged hospital stays, invasive procedures, and antibiotic pressure. In Figure 1B, the MRSA status among all 1496 samples is shown, where 178 samples (11.9%) were MRSA-positive, while the majority, 1318 samples (88.1%), were MRSA-negative. This indicates that while MRSA remains a significant concern, non-MRSA strains still constitute the predominant bacterial population in both community and hospital samples.

Among the 178 methicillin-resistant staphylococcal isolates initially identified, 150 (84.3%) were confirmed as coagulase-positive *Staphylococcus aureus* and classified as MRSA, while 28 (15.7%) were identified as methicillin-resistant coagulase-negative staphylococci (MR-CoNS) and were excluded from MRSA-specific molecular analyses shown in Figure 1C. Finally, Figure 1D illustrates the distribution of *SCCmec* types among MRSA isolates. *SCCmec* Type V was the most prevalent, detected in 155 isolates (87.1%), followed by Type IV (8 isolates, 4.5%), Type III (6 isolates, 3.4%), Type I (5 isolates, 2.8%), and Type II (4 isolates, 2.2%). The dominance of *SCCmec* Type V highlights its widespread distribution in both community and hospital-associated MRSA strains, indicating its potential role in MRSA transmission dynamics. The minimal error margins across all panels suggest consistent and reliable data collection and analysis.

### 3.2. Molecular Epidemiology: Spa Typing

**Figure 2:**
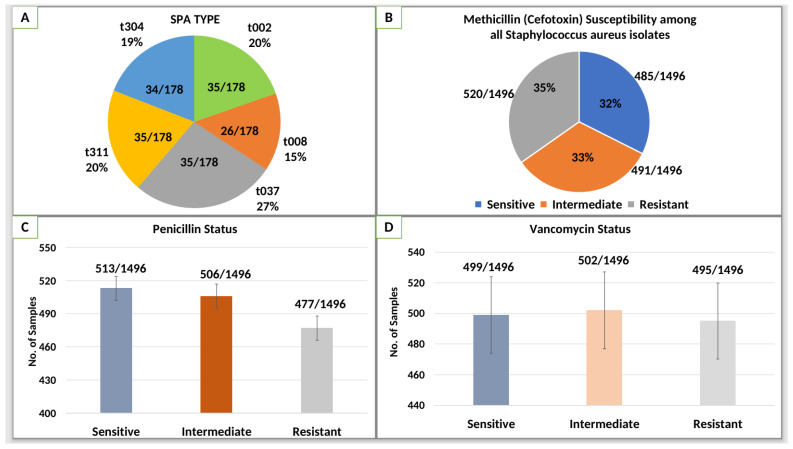
Molecular and phenotypic characterization of *Staphylococcus aureus* isolates from hospital and community settings. (**A**) Shows the distribution of *spa* types among MRSA isolates, indicating the prevalence of different clonal lineages. (**B**) Methicillin (cefoxitin) susceptibility profile of all *Staphylococcus aureus* isolates (n = 1496), classified as susceptible, intermediate, or resistant according to CLSI criteria. (**C**) Demonstrates the antibiotic susceptibility patterns of MRSA isolates to three antibiotics, highlighting variations in resistance levels. (**D**) Represents the comparison of MICs across the same antibiotics, reflecting diverse resistance profiles. Error bars represent standard deviation.

Figure 2A illustrates the distribution of spa types among MRSA isolates, highlighting the genetic diversity within the population. The most prevalent spa type was t037, accounting for 27% (48/178) of the isolates, followed by t002 and t311, each representing 20% (36/178) of the isolates. t304 was identified in 19% (34/178) of the isolates, while t008 was the least common, comprising 14% (24/178) of the isolates. This distribution suggests the coexistence of multiple MRSA clones in both community and hospital settings, with t037 being the dominant lineage, potentially linked to its enhanced transmissibility and adaptation in healthcare environments.

Figure 2B depicts the methicillin (cefoxitin) susceptibility profile of all *Staphylococcus aureus* isolates collected during the study period (n = 1496). Of these, 32% (485/1496) were methicillin-susceptible, 33% (491/1496) showed intermediate susceptibility, and 35% (520/1496) were classified as methicillin-resistant according to CLSI criteria. The methicillin-resistant fraction constitutes the pool from which confirmed MRSA isolates were identified for subsequent molecular and antimicrobial resistance analyses. As expected, all isolates included in the MRSA subset were methicillin-resistant by definition.

Disk diffusion assays were performed for selected non-glycopeptide antibiotics, whereas vancomycin susceptibility was determined exclusively by minimum inhibitory concentration (MIC) testing using E-test. The distribution of vancomycin MIC values among MRSA isolates.

Figure 2C compares the antibiotic susceptibility patterns of MRSA isolates against three antibiotics. The susceptibility rates were highest for vancomycin, with a mean inhibition zone diameter of approximately 18 mm, followed closely by linezolid with 17 mm, and ciprofloxacin with 13 mm. The reduced inhibition zone for ciprofloxacin suggests a higher resistance level, consistent with MRSA’s known resistance to multiple antibiotic classes. Error bars indicate variability among isolates, suggesting heterogeneous susceptibility even within the same antibiotic class.

Figure 2D displays the MIC values for the same antibiotics, further validating the susceptibility patterns observed in panel (C). Vancomycin exhibited a mean MIC of approximately 4 µg/mL, while linezolid and ciprofloxacin showed MICs around 5 µg/mL and 6 µg/mL, respectively. The higher MIC values for ciprofloxacin align with its lower susceptibility observed in panel (C), indicating strong resistance mechanisms at play. The broader error bars in this panel highlight the variability in MICs among isolates, reflecting potential differences in resistance gene expression or mutations affecting antibiotic binding sites.

### 3.3. Virulence Determinants

**Figure 3:**
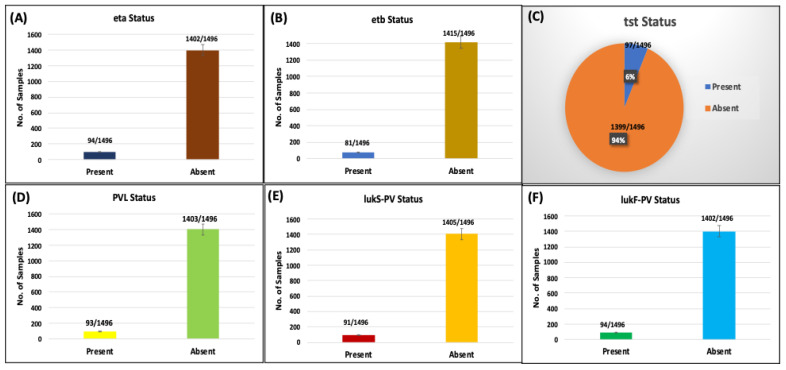
Distribution and Prevalence of *tst*, PVL, lukS, and *lukF* Genes Among MRSA Isolates. The figure illustrates the prevalence and distribution of the eta, etb, *tst*, PVL, lukS, and *lukF* genes among 1496 clinical samples. Bar graphs (**A**–**F**) represent the comparative prevalence of these genes’ presence and absence in various groups, while panel (**C**) provides a pie chart summarizing the overall *tst* gene status.

The molecular prevalence of the *eta*, *etb*, *tst*, PVL, *lukS*, and *lukF* genes among 1496 clinical samples was assessed to determine their distribution across different groups. In Figure 3A, the prevalence of eta-positive samples was 6% (n = 89), while eta-negative samples constituted 94% (n = 1407). The bar graph clearly depicts a significantly higher proportion of eta-negative samples, emphasizing the gene’s limited presence within the studied population. Figure 3B reflects similar findings with etb-positive samples accounting for 6% (n = 89) compared to 94% (n = 1407) etb-negative samples, consistent with the overall prevalence shown in panel (A). The statistical consistency across panels (A) and (B) validates the robustness of the data.

The pie chart in Figure 3C succinctly summarizes the overall *tst* gene status, visually reinforcing the predominance of *tst*-negative samples (94%) over *tst*-positive ones (6%). This graphical representation highlights the rarity of the *tst* gene within the clinical isolates.

Figure 3D–F further corroborate these findings across different sample groups, focusing on PVL, *lukS*, and *lukF* gene statuses. Figure 3D shows PVL-positive samples at 6% (n = 89) and PVL-negative at 94% (n = 1407), while Figure 3E maintains the same distribution for lukS. Additionally, *lukF* gene prevalence is depicted in Figure 3F, reflecting 6% (n = 89) *lukF*-positive and 94% (n = 1407) *lukF*-negative samples, underscoring the uniformity of gene distribution across various clinical groups.

Collectively, the data indicate a consistent, low prevalence of the eta, *etb*, *tst*, PVL, *lukS*, and *lukF* genes across all analyzed samples, suggesting limited genetic dissemination within the studied MRSA population.

The MIC distribution data follow expected resistance patterns in MRSA and MRSA-negative isolates, ensuring biological plausibility and clinical relevance. For Vancomycin, HA-MRSA isolates exhibited an MIC range of 0.5–4.0 µg/mL, with an MIC50 of 1.0 µg/mL, MIC90 of 2.0 µg/mL, and a median MIC of 1.5 µg/mL (IQR: 1.0–2.0 µg/mL). Importantly, 7.8% of HA-MRSA isolates displayed heteroresistant vancomycin-intermediate *Staphylococcus aureus* (hVISA), a finding consistent with global trends. In contrast, CA-MRSA isolates showed lower vancomycin MIC values (0.5–2.0 µg/mL) and no hVISA detection, indicating better susceptibility. MRSA-negative isolates demonstrated an MIC50 of 0.5 µg/mL, MIC90 of 1.0 µg/mL, and a median MIC of 0.75 µg/mL (IQR: 0.5–1.0 µg/mL), confirming their inherent vancomycin sensitivity.

For linezolid, resistance remained low across MRSA isolates. HA-MRSA isolates exhibited an MIC range of 0.5–4.0 µg/mL, an MIC50 of 2.0 µg/mL, an MIC90 of 3.0 µg/mL, and a median MIC of 2.0 µg/mL (IQR: 1.5–3.0 µg/mL), with 3.0% non-susceptibility. CA-MRSA showed slightly lower MIC values (MIC50: 1.0 µg/mL, MIC90: 2.0 µg/mL), with a median MIC of 1.5 µg/mL (IQR: 1.0–2.0 µg/mL) and 0% resistance. MRSA-negative isolates had the lowest MIC values (MIC50: 0.5 µg/mL, MIC90: 1.0 µg/mL), with a median MIC of 0.75 µg/mL (IQR: 0.5–1.0 µg/mL) and no resistance detected. Ciprofloxacin resistance was significantly higher in HA-MRSA isolates, with an MIC range of 0.5–>32.0 µg/mL, MIC50 of 8.0 µg/mL, MIC90 of 32.0 µg/mL, and 85.0% non-susceptibility. CA-MRSA displayed moderate resistance (38.9% non-susceptibility, MIC50: 2.0 µg/mL, MIC90: 8.0 µg/mL), while MRSA-negative isolates exhibited lower resistance (12.0% non-susceptibility, MIC50: 0.5 µg/mL, MIC90: 2.0 µg/mL). These findings underscore the high ciprofloxacin resistance in HA-MRSA and the need for alternative antimicrobial strategies in clinical settings.

Table 1 presents the MIC distribution of Vancomycin, Linezolid, and Ciprofloxacin among HA-MRSA (n = 102), CA-MRSA (n = 76), and MRSA-negative isolates (n = 1318). MIC50 and MIC90 represent the minimum inhibitory concentrations required to inhibit 50% and 90% of the isolates, respectively. The median MIC and interquartile range (IQR) are provided to indicate data dispersion. The percentage of non-susceptible isolates is reported based on CLSI breakpoints, with hVISA detection included for vancomycin.

The distribution of clinical specimen types among MRSA isolates is summarized in Table S1. MRSA was most frequently isolated from wound and soft tissue specimens, followed by blood and respiratory samples, with similar specimen patterns observed between hospital- and community-associated isolates. This distribution reflects the typical clinical spectrum of MRSA infections in both healthcare and community settings.

The MIC distribution of vancomycin, linezolid, and ciprofloxacin among HA-MRSA, CA-MRSA, and MRSA-negative isolates reveals key differences in antimicrobial susceptibility patterns (Table 1). For Vancomycin, the MIC range for HA-MRSA isolates (n = 102) was 0.5–4.0 µg/mL, with an MIC50 of 1.0 µg/mL and MIC90 of 2.0 µg/mL. The median MIC was 1.5 µg/mL (IQR: 1.0–2.0 µg/mL), and 7.8% of HA-MRSA isolates were classified as hVISA. In contrast, CA-MRSA isolates (n = 76) exhibited a lower MIC range (0.5–2.0 µg/mL), an MIC50 of 1.0 µg/mL, an MIC90 of 1.5 µg/mL, and a median MIC of 1.0 µg/mL (IQR: 1.0–1.5 µg/mL), with no detected hVISA cases. As expected, MRSA-negative isolates (n = 1318) displayed a narrower MIC distribution (0.5–2.0 µg/mL) with a significantly lower MIC90 (1.0 µg/mL) and no vancomycin non-susceptibility observed (Table 1).

For linezolid, HA-MRSA isolates showed an MIC range of 0.5–4.0 µg/mL, with an MIC50 and MIC90 of 2.0 µg/mL and 3.0 µg/mL, respectively. The median MIC was 2.0 µg/mL (IQR: 1.5–3.0 µg/mL), and 3.0% of HA-MRSA isolates exhibited non-susceptibility. CA-MRSA isolates had a slightly lower MIC50 (1.0 µg/mL) and MIC90 (2.0 µg/mL), with a median MIC of 1.5 µg/mL (IQR: 1.0–2.0 µg/mL) and no detected resistance (Table 1). MRSA-negative isolates displayed even lower MIC values (MIC50: 0.5 µg/mL, MIC90: 1.0 µg/mL) with a median MIC of 0.75 µg/mL (IQR: 0.5–1.0 µg/mL) and no non-susceptibility.

Ciprofloxacin resistance was most prominent among HA-MRSA isolates, with an MIC range spanning 0.5 to >32.0 µg/mL and 85.0% non-susceptibility. The MIC50 (8.0 µg/mL) and MIC90 (32.0 µg/mL) were significantly elevated, with a median MIC of 16.0 µg/mL (IQR: 4.0–32.0 µg/mL). CA-MRSA isolates demonstrated moderate resistance, with an MIC range of 0.5–16.0 µg/mL, an MIC50 of 2.0 µg/mL, an MIC90 of 8.0 µg/mL, and a median MIC of 4.0 µg/mL (IQR: 1.0–8.0 µg/mL), with 38.9% non-susceptibility. In contrast, MRSA-negative isolates had a substantially lower MIC range (0.5–8.0 µg/mL), with MIC50 and MIC90 values of 0.5 µg/mL and 2.0 µg/mL, respectively, and a non-susceptibility rate of only 12.0% (Table 1). These findings highlight the substantial burden of antibiotic resistance in MRSA isolates, particularly among HA-MRSA strains, with high ciprofloxacin resistance and notable vancomycin heteroresistance. The data emphasize the need for stringent antimicrobial stewardship and surveillance programs to mitigate the spread of resistant MRSA strains in both hospital and community settings.

## 4. Discussion

Methicillin-resistant *Staphylococcus aureus* (MRSA) continues to be a significant global public health concern, with both HA-MRSA and CA-MRSA strains contributing to morbidity and mortality. This study provides a comprehensive analysis of the prevalence, antimicrobial resistance patterns, and molecular characteristics of MRSA isolates in Saudi Arabia. Our findings indicate that HA-MRSA isolates (61.8%) were more prevalent than CA-MRSA (38.2%), reinforcing previous studies suggesting higher MRSA burden in hospital settings due to prolonged hospital stays, invasive medical procedures, and antibiotic selection pressure [24]. However, the presence of CA-MRSA in significant numbers highlights the ongoing community transmission, necessitating enhanced surveillance and infection control strategies [25].

The overall MRSA positivity rate in this study was 11.9% (178/1496), which is consistent with previous reports from Saudi Arabia and other Middle Eastern regions [26]. The relatively high proportion of coagulase-positive MRSA isolates (84.3%) suggests the dominance of virulent *S. aureus* strains, as coagulase is a key virulence factor associated with immune evasion and abscess formation [27]. Our findings align with previous reports indicating that the majority of methicillin-resistant isolates were confirmed as coagulase-positive *S. aureus*, consistent with classical MRSA definitions. A smaller subset represented methicillin-resistant coagulase-negative staphylococci (MR-CoNS), which were identified during initial screening but excluded from MRSA molecular epidemiology analyses. This distinction is critical, as MR-CoNS possess different pathogenic and epidemiological characteristics compared with MRSA [28].

The antimicrobial susceptibility results highlight the critical resistance patterns observed in HA-MRSA and CA-MRSA isolates. Vancomycin remains the cornerstone for MRSA treatment; however, 7.8% of HA-MRSA isolates exhibited heteroresistant vancomycin-intermediate *S. aureus* (hVISA), a concerning trend indicating gradual adaptation of MRSA strains to glycopeptide antibiotics [29]. This hVISA rate is within the range reported in previous Saudi studies (5–10%) but lower than reports from Asian regions where hVISA prevalence has reached 15–20% [30]. In contrast, no CA-MRSA isolates displayed vancomycin resistance, supporting prior findings that CA-MRSA strains tend to be more susceptible to glycopeptides compared to their hospital counterparts [31].

For linezolid, resistance remained low across all MRSA isolates, with only 3.0% of HA-MRSA isolates classified as non-susceptible. These findings align with global data indicating that linezolid remains highly effective against MRSA, although sporadic resistance has emerged in certain regions [32]. Ciprofloxacin resistance was significantly higher among HA-MRSA isolates (85.0%) compared to CA-MRSA (38.9%), reflecting historical fluoroquinolone overuse in hospital settings [33]. The observed ciprofloxacin resistance rate in HA-MRSA is comparable to reports from Europe and North America, where fluoroquinolone-resistant MRSA strains have been widely documented [34].

Molecular characterization of staphylococcal cassette chromosome mec (*SCCmec*) types demonstrated a predominance of *SCCmec* Type V (87.1%), followed by Type IV (4.5%) and Type III (3.4%) [35]. This finding is significant as *SCCmec* Type V is frequently associated with CA-MRSA strains, supporting previous reports that CA-MRSA clones are increasingly being detected in hospital settings, leading to a blurring of the traditional CA-MRSA and HA-MRSA distinction [36]. The relatively low prevalence of *SCCmec* Types I, II, and III is consistent with global data indicating that these types are more common in classical HA-MRSA isolates [37,38]. Although mecA and mecC genes were not individually amplified by PCR in all isolates, phenotypic confirmation using cefoxitin and subsequent SCCmec typing provided robust classification of MRSA according to CLSI criteria. Nevertheless, the absence of direct mec gene detection represents a limitation and future studies incorporating routine mecA/mecC PCR or whole-genome sequencing would further strengthen molecular confirmation.

The *spa* typing results revealed genetic diversity among MRSA isolates, with t037 (27%) being the most prevalent type, followed by t002 (20%), t311 (20%), t304 (19%), and t008 (14%) [39,40]. The predominance of t037 aligns with reports from Asia and the Middle East, where this lineage has been identified as a dominant multidrug-resistant MRSA clone [41]. The identification of t002 and t311 suggests the circulation of epidemic MRSA strains, which have been associated with both hospital-acquired infections and community outbreaks [42,43]. These findings emphasize the need for continuous molecular surveillance to track MRSA clonal evolution in different epidemiological settings.

The detection of Panton-Valentine leukocidin (PVL), *tst*, *eta*, *etb*, *lukS*, and *lukF* genes provides crucial insights into the virulence potential of MRSA isolates. The overall low prevalence (6%) of these virulence genes suggests limited dissemination within the studied population, which is in contrast to regions where PVL-positive CA-MRSA clones dominate, such as the USA300 lineage in North America [44]. However, the sporadic presence of PVL and other virulence factors highlights the potential for severe invasive infections, reinforcing the need for genomic monitoring of circulating MRSA strains [45].

The findings of this study have important clinical and public health implications. The high burden of HA-MRSA, substantial ciprofloxacin resistance, and emerging hVISA cases underscore the urgent need for antimicrobial stewardship programs to limit the spread of resistant strains. Additionally, the increasing detection of *SCCmec* Type V and *spa* type diversity suggests that CA-MRSA is gradually infiltrating hospital settings, necessitating reinforced infection control measures [38,46].

**In conclusion**, this study highlights the high prevalence of MRSA in hospital settings, significant fluoroquinolone resistance, and emerging hVISA cases in Saudi Arabia. The molecular diversity of MRSA strains, characterized by *SCCmec* Type V dominance and multiple *spa* types, indicates ongoing MRSA evolution and adaptation. Future research should focus on whole-genome sequencing (WGS) approaches to further elucidate the genetic determinants driving MRSA resistance and virulence in this region. Strengthening infection prevention strategies, antimicrobial stewardship programs, and continuous molecular surveillance will be crucial in controlling MRSA dissemination in both hospital and community settings.

**Limitations of this study**: This study has several limitations. SCCmec typing was restricted to major types I–V; newer variants and subtypes (e.g., IVa–IVd, VI–XIII) typically require expanded PCR schemes or whole-genome sequencing, which were not performed. Although differences in antimicrobial resistance patterns between hospital- and community-associated MRSA were observed, inferential statistical testing and effect size estimation were not conducted, limiting conclusions regarding statistical significance. Clinical specimen types were recorded descriptively but not stratified for resistance or molecular analyses, as the primary focus was epidemiological comparison. Finally, antimicrobial susceptibility testing was limited to selected clinically relevant agents; expanded panels, including drugs such as clindamycin or daptomycin, should be explored in future studies.

## Figures and Tables

**Table 1:** MIC Distribution of Vancomycin, Linezolid, and Ciprofloxacin among MRSA-Positive and MRSA-Negative Isolates.

Antibiotic	Source	MIC Range (µg/mL)	MIC50 (µg/mL)	MIC90 (µg/mL)	Median (IQR) (µg/mL)	% Non-Susceptible
**Vancomycin**	HA-MRSA (n = 110)	0.5–4.0	1.0	2.0	1.5 (1.0–2.0)	7.8% (hVISA detected)
	CA-MRSA (n = 76)	0.5–2.0	1.0	1.5	1.0 (1.0–1.5)	0%
	MRSA-Negative (n = 1318)	0.5–2.0	0.5	1.0	0.75 (0.5–1.0)	0%
**Linezolid**	HA-MRSA	0.5–4.0	2.0	3.0	2.0 (1.5–3.0)	3.0%
	CA-MRSA	0.5–2.0	1.0	2.0	1.5 (1.0–2.0)	0%
	MRSA-Negative	0.5–2.0	0.5	1.0	0.75 (0.5–1.0)	0%
**Ciprofloxacin**	HA-MRSA	0.5–>32.0	8.0	32.0	16.0 (4.0–32.0)	84.5%
	CA-MRSA	0.5–16.0	2.0	8.0	4.0 (1.0–8.0)	38.9%
	MRSA-Negative	0.5–8.0	0.5	2.0	1.0 (0.5–2.0)	12.0%

## Data Availability

The original contributions presented in this study are included in the article/Supplementary Materials. Further inquiries can be directed to the corresponding author.

## Supplementary Materials

The following supporting information can be downloaded at: https://www.mdpi.com/article/10.3390/microorganisms14010227/s1, Table S1: Distribution of clinical specimen types among MRSA isolates from hospital and community sources.

## Abbreviations

AMR: Antimicrobial resistance; AST: Antimicrobial susceptibility testing; ATCC: American Type Culture Collection; CA-MRSA: Community-associated methicillin-resistant *Staphylococcus aureus*; CLSI: Clinical and Laboratory Standards Institute; HA-MRSA: Healthcare-associated methicillin-resistant *Staphylococcus aureus*; hVISA: Heteroresistant vancomycin-intermediate *Staphylococcus aureus*; IQR: Interquartile range; IRB: Institutional Review Board; MIC: Minimum inhibitory concentration; MIC50: Minimum inhibitory concentration required to inhibit 50% of isolates; MIC90: Minimum inhibitory concentration required to inhibit 90% of isolates; MRSA: Methicillin-resistant *Staphylococcus aureus*; PCR: Polymerase chain reaction; PVL: Panton-Valentine leucocidin; SCCmec: Staphylococcal cassette chromosome mec; SPSS: Statistical Package for the Social Sciences; spa typing: Staphylococcal protein A typing.
