# Supplementary material for: Prevalence, Antimicrobial Resistance, and Molecular Characteristics of MRSA in Saudi Arabia: A Retrospective Study
Source: Microorganisms. 2026 Jan 19;14(1):227. doi: 10.3390/microorganisms14010227 (PMC12844393; doi:10.3390/microorganisms14010227)
Supplement: Supplementary file 1 [file microorganisms-14-00227-s001.zip › S1 Table.pdf]

Table S1: Distribution of clinical specimen types among MRSA isolates from hospital and community sources.

**Specimen type**	**Total MRSA (n = 178)**	**HA-MRSA (n = 110)**	**CA-MRSA (n = 68)**
Wound/Pus/SSTI	78 (43.8%)	40 (36.4%)	38 (55.9%)
Blood (bacteremia)	32 (18.0%)	26 (23.6%)	6 (8.8%)
Respiratory specimens	28 (15.7%)	22 (20.0%)	6 (8.8%)
Urine	2 (12.4%)	14 (12.7%)	8 (11.8%)
Other sterile sites	18 (10.1%)	8 (7.3%)	10 (14.7%)
Total	178 (100%)	110 (100%)	68 (100%)
